# Ectomycorrhizal fungal communities in natural and urban ecosystems: *Quercus humboldtii* as a study case in the tropical Andes

**DOI:** 10.1007/s00572-024-01140-0

**Published:** 2024-03-14

**Authors:** Juan David Sanchez-Tello, Adriana Corrales

**Affiliations:** 1 Center for Research in Microbiology and Biotechnology-UR (CIMBIUR), Faculty of Natural Sciences, Universidad del Rosario, Bogotá, Colombia; 2 Society for the Protection of Underground Networks, SPUN, 3500 South DuPont Highway, Dover, DE 19901 USA

**Keywords:** Ectomycorrhizal fungi, Community structure, *Quercus Humboldtii* Bonpl., Rural vs urban communities, Andean oak

## Abstract

Worldwide urban landscapes are expanding because of the growing human population. Urban ecosystems serve as habitats to highly diverse communities. However, studies focusing on the diversity and structure of ectomycorrhizal communities are uncommon in this habitat. In Colombia, *Quercus humboldtii* Bonpl. is an ectomycorrhizal tree thriving in tropical montane forests hosting a high diversity of ectomycorrhizal fungi. *Q. humboldtii* is planted as an urban tree in Bogotá (Colombia). We studied how root-associated fungal communities of this tree change between natural and urban areas. Using Illumina sequencing, we amplified the ITS1 region and analyzed the resulting data using both OTUs and Amplicon Sequence Variants (ASVs) bioinformatics pipelines. The results obtained using both pipelines showed no substantial differences between OTUs and ASVs for the community patterns of root-associated fungi, and only differences in species richness were observed. We found no significant differences in the species richness between urban and rural sites based on Fisher’s alpha or species-accumulation curves. However, we found significant differences in the community composition of fungi present in the roots of rural and urban trees with rural communities being dominated by *Russula* and *Lactarius* and urban communities by *Scleroderma*, *Hydnangium*, and *Trechispora*, suggesting a high impact of urban disturbances on ectomycorrhizal fungal communities. Our results highlight the importance of urban trees as reservoirs of fungal diversity and the potential impact of urban conditions on favoring fungal species adapted to more disturbed ecosystems.

**Supplementary Information:**

The online version contains supplementary material available at 10.1007/s00572-024-01140-0.

## Introduction

The richness and abundance of tropical Fagales and their associated ectomycorrhizal (ECM) fungi usually peak in montane forests. These forests are at medium elevations and have high levels of precipitation, intermediate temperatures, and high density of ECM host trees (Corrales et al. 2018). Because of their enzymatic capabilities and high growth rates, ECM fungi can control some ecosystem processes including soil carbon storage and soil nutrient availability (Corrales et al. 2018; Vasco-Palacios et al. 2020). However, the ecology and ecosystem function of tropical ECM fungi are not fully understood (Corrales et al. 2022a).

Urban landscapes are becoming more important as a habitat for wild species because of a growing human population and an increasing urbanization. An important part of these urban landscapes are urban ecosystems, consisting of areas with vegetation, such as parks, gardens, and road separators found in cities (Stevenson et al. 2020). Among other organisms, these ecosystems serve as habitat to plant and fungal communities. However, urban ecosystems often suffer from suboptimal environmental factors such as soil compaction, air pollution, and limited space for root development (Olchowik et al. 2020). Additionally, other human interventions can alter fungal community composition and structure directly, influencing plant communities or soil through fertilization or watering (Baruch et al. 2020). Few studies have focused on the structure of fungal communities in urban ecosystems, including those ECM fungal communities from temperate regions (Stevenson et al. 2020).

Soil variables, such as levels of phosphorus (P), nitrogen (N), and N:P ratio, are factors that can affect soil fungal communities. A study by Baruch et al. (2020), on the composition and structure of soil fungal communities within different urban ecosystems (sports fields, community gardens, parklands, and young/old revegetation) in Australia, found that soil fungal diversity and community structure differed among urban ecosystems because of differences in soils, vegetation, and management practices. In addition, a study focusing on ECM fungal communities associated with *Quercus spp*. in rural and urban sites in Manhattan (Kansas, USA) found that edaphic variables like P, levels of heavy metals, and organic matter were the most influential factors in the composition of the ECM communities (Jumpponen et al. 2010). Also, Olchowik et al. (2020) in a study in Warsaw (Poland) found that ECM fungal richness did not differ between healthy trees growing in parks or street habitats. However, they found that healthy trees growing in streets and parks have a significantly higher ECM richness and colonization than unhealthy trees growing in the same streets. They also found that concentrations of Na, Cl, and Pb in the soil have a negative effect on the ECM fungal colonization (Olchowik et al. 2020).

*Quercus* is a genus with approximately 500 species of trees and shrubs with a global distribution, mainly in the temperate northern hemisphere, being a conspicuous member of the forests of North America, Europe, and Asia (Manos et al. 1999). This genus has great ecological and economic importance because it is a source of food and habitat for wildlife and can have socio-economic benefits such as the provision of wood and food for human communities (Aldrich and Cavender-Bares 2011; Pagano and Lugo 2019). *Quercus* forms associations with ECM fungi that contribute to the plant’s nutrition and intervene in soil biogeochemical processes. In Colombia, *Quercus humboldtii* Bonpl. is present in montane forests and is the only native species of this genus (Rangel and Avella 2011). It is the most abundant ECM host tree in the country (Rangel and Avella 2011).

There are few studies focusing on natural *Quercus* ECM fungal communities in the tropics (García-Guzmán et al. 2017; Morris et al. 2008; Waring et al. 2016). In dry tropical forests dominated by *Quercus* spp. in Costa Rica, Desai et al. (2016) found a high diversity of ECM fungi, dominated by Russulaceae and Thelephoraceae and a low prevalence of Ascomycota. These results are similar to those obtained by Morris et al. (2008) who found that the ECM fungal communities in a tropical forest in Mexico were dominated by the families Russulaceae, Cortinariaceae, Inocybaceae, and Thelephoraceae. For *Quercus humboldtii* specifically, several studies based on fruiting body collection in Colombia had shown this species hosts a high diversity of ECM fungi. From the 172 species of ECM fungi reported for Colombia, 116 are associated with *Quercus* monodominant forest (Pagano and Lugo 2019; Peña-Venegas and Vasco-Palacios 2019). The most species-rich lineages of ECM fungi reported for *Q. humboldtii* forests are Boletaceae, Amanitaceae, and Russulaceae (Pagano and Lugo 2019; Peña-Venegas and Vasco-Palacios 2019; Vargas and Restrepo 2020; Corrales et al. 2022b).

Due to its beauty and ecological importance, *Q. humboldtii* is commonly used as an urban tree in Colombia. Even if not many studies have focused on the specific *Q. humboldtii* resistance to environmental stressors of Bogotá (pollutants, soils, etc.), it grows well under urban conditions. Lamilla et al. (2022) found an 80% prevalence of phytoplasma infection in Bogotá’s oaks, but without having lethal or severe consequences to the tree, suggesting a high level of resistance in these environments. The ECM communities associated with this species have not yet been studied in urban ecosystems. This study explores the community composition of root-associated fungi of *Quercus humboldtii* (Fagaceae) using high-throughput amplicon sequencing of the fungal ITS1 region in urban and natural ecosystems. We aim to determine how the composition of the ECM fungal communities associated with *Q. humboldtii* changes with biotic and abiotic variables. Because of gradients of air pollutants, changes on soil variables as a result of application of fertilizers and presence of pets, and other differences in cultivation techniques of urban trees, we expect to find shifts in the composition of ECM fungal species associated with *Q. humboldtii* between rural and urban ecosystem and also among urban sites. We also hypothesize that fungal communities from urban ecosystems will have a greater abundance of taxa adapted to high levels of N pollution.

## Materials and methods

### Study area

**Fig. 1:**
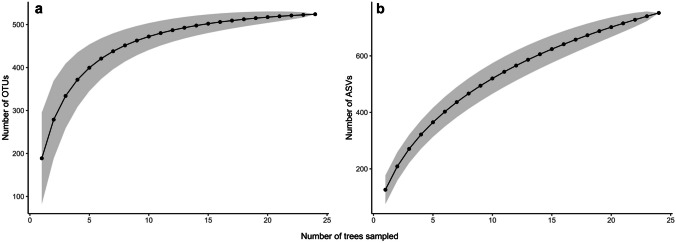
Species accumulation curves including all 24 trees sampled in urban and rural sites for **a** OTUs and **b** ASVs

We selected two study sites located in the Andean Mountain range of Colombia in the Cundinamarca province (Fig. 1S). The first site was the Chicaque Natural Reserve (4°36′22″ N, 74°18′17″ W) located in the eastern Andean cordillera. The reserve has an extension of 308.88 ha approx., and it is part of the La Playa watershed, located between the municipalities of Soacha and San Antonio (Colparques 2023; Bernal and Guevara 2019). The reserve presents an altitudinal gradient from 2000 to 2720 m.a.s.l., with an average temperature of 14.5 °C, and a bimodal rainfall regime with the highest rainfall between March–May and October–November with an annual precipitation of ~ 2000 mm (Colparques 2023; Rivera and Córdoba 1998). The reserve has a natural *Q. humboldtii* monodominant forest which is characterized by soils with a deep organic horizon (Colparques 2023), and it was considered as a rural sampling site for this study.

The second study site was the city of Bogotá; specifically, sampling was performed in the districts of Puente Aranda (4°36′45″ N, 74°06′24″ W; 4°36′09″ N, 74°06′55″ W) and Teusaquillo (4°40′14″ N, 74°5′35″ W). Bogotá has 7,181,469 inhabitants and is located at an average altitude of 2640 m.a.s.l (DANE 2019). The average temperature is 14 °C, and the annual rainfall distribution has a bimodal regime with an average annual rainfall of 840 mm (IDIGER 2023). The district of Puente Aranda has a higher incidence of air pollutants with a monthly average of 20.24 µg/m^3^ for NO_2_, 17.35 µg/m^3^ for PM2.5, and of 40.08 µg/m^3^ for PM10, while in Teusaquillo, the monthly averages are slightly lower with 17.93 µg/m^3^ for NO_2_, 15.16 µg/m^3^ for PM2.5, and 25.9 µg/m^3^ for PM10 (SISAIRE 2021).

In Teusaquillo, the sampled parks are open to the public and the entry of pets is allowed. Street divider samples taken in this locality were collected next to the highway Avenue 50 (AV), with high vehicular traffic that produces high levels of atmospheric pollutants like CO, O_3_, SO_2_, and NO_2_ (SISAIRE 2021). In the district of Puente Aranda, three of the urban parks sampled were parks where animals are allowed to enter (CSM, PCR, PLC), and from Ciudad Montes Park (PM), a park that has restricted hours for visitors, surveillance, tree maintenance, and pets are not allowed.

### Root and soil collection

A total of 24 individuals of *Q. humboldtii* were sampled, four were sampled in Chicaque (rural area) and twenty were sampled in Bogotá (urban area, specifically five in Avenue 50, seven in Teusaquillo, and eight in Puente Aranda). This sampling was carried out during the COVID-19 lockdowns, and therefore, it was not possible to obtain more samples for the natural area. Ectomycorrhizal roots of the selected oaks were excavated up to 1 m from the trunk until finding fine roots, making sure that they belonged to the target tree. Roots were stored in plastic bags and refrigerated after collection. Each sample was carefully cleaned with distilled water under stereoscope and cut into 2 cm long pieces; 10 pieces (20 cm) of root samples per tree were stored in 2% CTAB buffer in −20 °C until DNA extraction.

In addition, soil samples were collected under each sampled tree and analyzed for the following variables: texture (% of sand, silt and clay), pH, organic C, available phosphorus (P, mg/kg), cation exchange capacity (CEC), effective cation exchange capacity (ECEC), Mg (cmol(+)/kg), Na (cmol(+)/kg), K (cmol(+)/kg), Ca (cmol(+)/kg, C:N ratio, base saturation percentage (SB), total bases (B.T), exchangeable acidity, and exchangeable acidity saturation percent (SAI) in the National Laboratory of Soils of the Instituto Geográfico Agustín Codazzi (Bogotá, Colombia).

### DNA extraction and amplification

The DNA extraction of the samples was done using the cetyl-trimethylammonium bromide (CTAB) 2% protocol following Gardes and Bruns (1993). The ITS1 region was PCR-amplified and sequenced on Illumina HiSeq2500 PE250 by Novogene Bioinformatics Technology Co. Ltd. (Beijing, China), using the fungal specific primers ITS5-1737 F (GGAAGTAAAAGTCGTAACAAGG) and ITS2-2043R (GCTGCGTTCTTCATCGATGC). High throughput sequences are available in Sequence Read archive (SRA) under accession PRJNA1063376.

### Bioinformatics

Sequences were processed with two different pipelines using OTUs (operational taxonomic units) and ASVs (amplicon sequence variants) to compare how different bioinformatic tools influence community diversity metrics. For the OTUs pipeline, the Illumina data were analyzed using AMPtk v1.3.0 (Palmer et al. 2018) following the previously published protocol by Corrales et al. (2020). In brief, the reads were demultiplexed following the documentation established by AMPtk 1.3.0. (https://amptk.readthedocs.io/en/latest/pre-processing.html), and sequences with a length less than 150 bp were discarded. Sequence reads were clustered at 97% similarity to generate operational taxonomic units (OTUs). OTUs were assigned to taxonomic classifications using the taxonomy algorithm in AMPtk (Palmer et al. 2018) against the UNITE database Version 02.04.2020 (Abarenkov et al. 2020). All non-fungal OTUs and OTUs with fewer than 10 sequences were excluded from the dataset. Furthermore, the ecological guild of each OTU per site was determined using the FungalTraits database (Põlme et al. 2020) based on their genus classification.

For the ASVs bioinformatics, the Illumina Mi-Seq data was processed using DADA2 version 1.16.0 package (Callahan et al. 2016) in R version 4.0.4 (R Core Team 2020) following the DADA2 ITS Pipeline Workflow (https://benjjneb.github.io/dada2/ITS_workflow.html). The primers were identified and subsequently removed using *cutadapt* v3.2 (Martin 2011). Subsequently, the filterAndTrim function of DADA2 was used with the following parameters (maxN = 0, maxEE = 2, truncQ = 2, minLen = 50) and a read length between 224 and 429 bp. Sequence error and dereplication rates were performed using the *learnErrors* and *derepFastq* functions, respectively, using the default parameters. Chimeras were removed using the *removeBimeraDenovo* function, applying the consensus method. Taxonomy was assigned against the UNITE database Version 02.04.2020 (Abarenkov et al. 2020) with the dynamic grouping thresholds, using the Naïve Bayesian Classifier (Wang et al. 2007) implemented in the *assignTaxonomy* DADA2 function. In this step, the taxonomy levels from phylum to genus were assigned. All taxa other than fungi and with less than 10 sequences were excluded from the data set. Finally, the ecological guild of each ASV per site was determined using the FungalTraits database (Põlme et al. 2020) based on their genus classification.

### Statistical analyses

Statistical tests were carried out in R v4.0.4 (R Core Team 2020). The soil variables were analyzed using a Kruskal-Wallis test looking for differences between sites. Alpha diversity was calculated by site using Shannon and Fisher indices using the phyloseq R package v1.30 (McMurdie and Holmes 2013). The alpha diversity was compared among sites using ANOVA and checked for normality using the Shapiro-Wilk test. Species accumulation curves were performed for OTUs and ASVs using the Vegan v2.5-7 (Oksanen et al. 2017) in R. We also constructed abundance bar plots and heatmaps using genus and family level relative abundance per site using the phyloseq package in R.

To compare fungal community species composition among sites, a nonmetric multidimensional scaling (NMDS) was performed for the following groups: (1) Chicaque, (2) Avenue 50, (3) Puente Aranda parks, and (4) Teusaquillo parks. In addition, we run an ADONIS analysis to test statistical differences in community compositions among sites using Bray-Curtis Dissimilarity and 1000 permutations (Anderson et al. 2011; Oksanen et al. 2008). After the ADONIS analysis, the soil variables that had a statistically significant effect on the structure of the communities were added to the NMDS. The results were considered significant at *p* < 0.05. The urban soil variables were compared among sites using ANOVA.

## Results

**Fig. 2:**
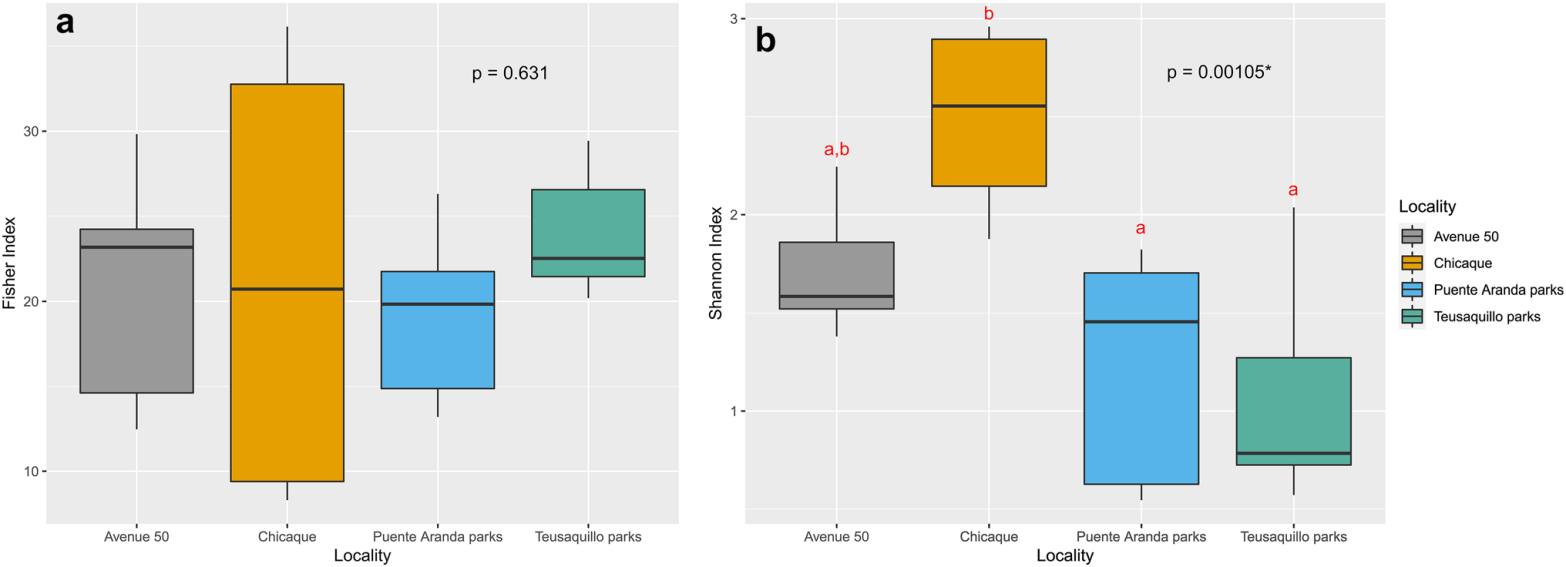
Alpha diversity indices boxplots for OTUs for urban (Avenue 50, Puente Aranda parks, Teusaquillo parks) and rural (Chicaque) sites. **a** The Alpha Fisher diversity index, **b** Shannon index. Avenue 50 (*n* = 5), Puente Aranda parks (*n* = 8), Teusaquillo parks (*n* = 7), Chicaque (*n* = 4)

A total of 4,778,648 valid reads were obtained with an average of 230,583 reads per sample. A total of 735,516 reads were recovered for Chicaque and 4,043,132 reads from Bogotá. Total number of OTUs was 1154, and 4,687,262 reads were assigned to OTUs (98%). We found 949 OTUs in Bogotá and 514 OTUs in Chicaque. Almost all soil variables show significant differences among sites (Table 1; Fig. 2S).

**Table 1:** Mean and standard deviation (SD) of soil variables for urban (Avenue 50, Puente Aranda parks, Teusaquillo parks) and rural (Chicaque) sites

**Variables**	** Site **									
	**Avenue 50** **(*n*** **= 5)**		**Puente Aranda parks** **(*n*** **= 8)**		**Teusaquillo parks** **(*n*** **= 7)**		**Chicaque** **(*n*** **= 4)**		***p*-value**	
	Mean	SD	Mean	SD	Mean	SD	Mean	SD		
Sand %	63.6	7.4	59.1	7.8	65.1	8.4	53.9	4.0	0.06	
Clay %	9.8	2.2	11.3	3.3	8.9	2.9	13.1	3.4	0.11	
Silt %	26.6	6.7	29.6	9.7	26	7.3	37.5	6.6	0.52	
pH	5.1	0.5	5.0	0.4	4.5	0.6	4.2	0.2	0.01	*
Organic C %	6.7	1.5	6.1	1.9	5.8	1.3	8.6	8.9	0.70	
Total C %	9.1	2.1	8.3	2.5	7.8	1.7	14.8	15.3	0.71	
N %	0.8	0.2	0.7	0.2	0.7	0.2	1.5	1.5	0.71	
C:N	0.2	0.1	11.6	0.1	11.6	0	10.1	0.2	0.01	*
P (mg/kg)	83.5	57.3	234.8	343	135	90.5	6.8	1.4	0.02	*
CEC (cmol(+)/kg)	31.3	10.3	39.7	13	35.8	6.9	87.2	13.1	0.01	*
Mg (cmol(+)/kg)	0.7	0.2	1.5	0.8	0.7	0.4	0.4	0.1	0.01	*
Na (cmol(+)/kg)	0.3	0.3	0.4	0.5	0.5	0.7	0	0	0.01	*
Exchangeable acidity (cmol(+)/kg)	0.9	0.8	1.4	1.2	1.0	1.1	10.5	2.8	0.02	*
SAI %	12.2	11.8	13.3	11.4	17.8	18.9	92.6	1.7	0.02	*
ECEC (cmol(+)/kg)	8.5	2.7	11.1	5.7	6.0	2.5	11.2	2.8	0.05	*
Ca (cmol(+)/kg)	6.0	2.6	6.6	3.9	2.7	2.4	0	0	0.00	*
K (cmol(+)/kg)	0.7	0.3	1.2	0.4	1.1	0.3	0.4	0.0	0.01	*
B.T. (cmol(+)/kg)	7.6	3.0	9.7	5.3	5.0	2.7	0.8	0.1	0.00	*
SB %	28.7	18.6	27.1	14.8	14.9	8.9	0.9	0.1	0.01	*
Ca/Mg	9.8	5.2	4.7	2.8	3.6	1.7	0	0	0.0	*
Mg/K	1.2	0.7	1.2	0.5	0.7	0.5	1.1	0.1	0.12	
Ca/K	11.1	8.6	5.7	2.7	2.6	2.0	0	0	0.00	*
(Ca+Mg)/K	12.3	9.1	6.9	3.0	3.3	2.3	0	0	0.00	*

Variables that showed significant differences in the Kruskal-Wallis test are represented by a "*" (*p* < 0.05)

When comparing alpha diversity patterns between OTU and ASV pipelines, we found that species accumulation curves using OTUs reach a plateau indicating a sufficient sampling effort for the community (Fig. 1a). However, in the case of the ASVs, species accumulation curves did not reach a plateau indicating that more sampling effort was necessary to have a complete representation of species richness in the community (Fig. 1b). In function of our sampling effort, the Chao index for the ASVs estimated that only 752 ASVs were found from the 1034 that were expected.

**Fig. 3:**
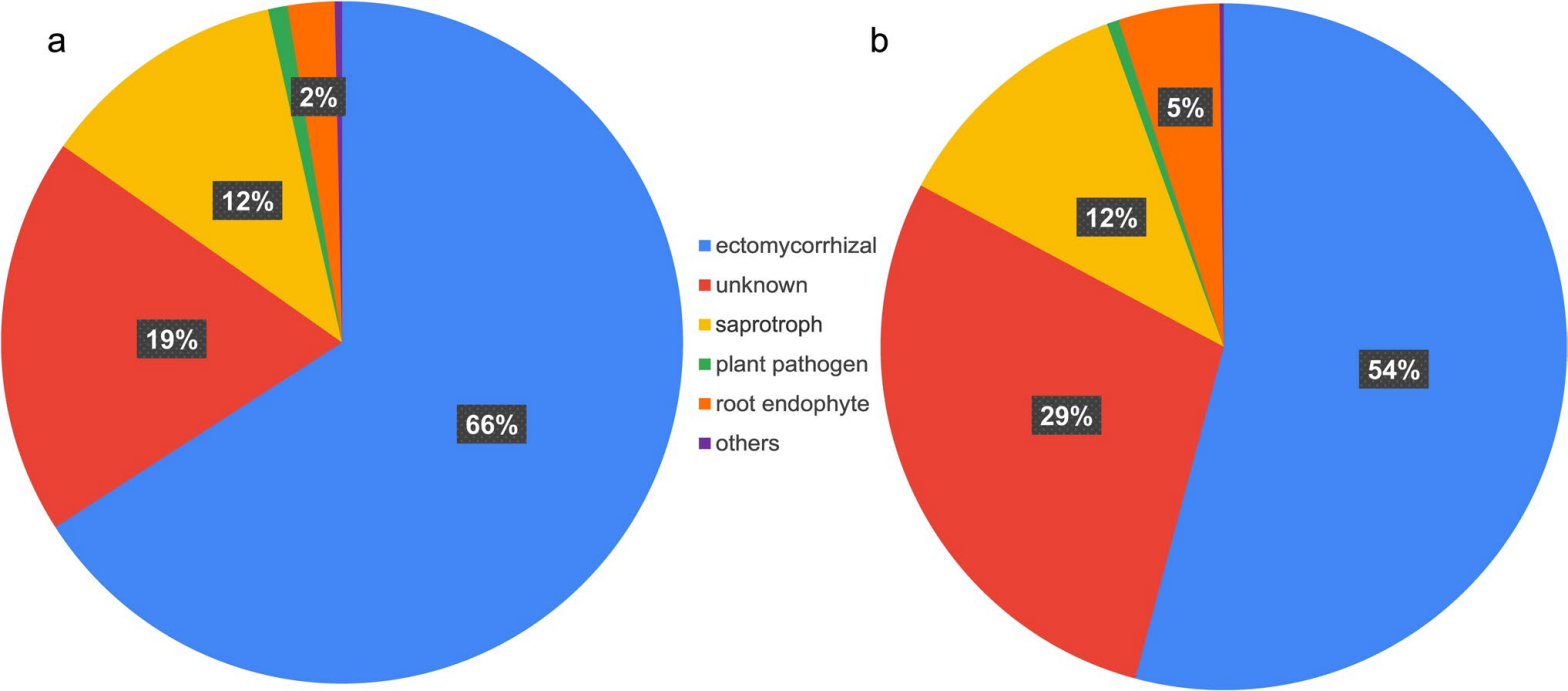
Classification of OTUs primary lifestyle classification based on the FungalTraits database for **a** urban samples and **b** rural samples

For both OTUs and ASVs, Alpha Fisher diversity index did not show significant differences among the four sampled areas (Figs. 2a and 3Sa). However, the Shannon index showed significantly lower species richness in the urban communities of Puente Aranda and Teusaquillo parks compared with the rural site of Chicaque (Figs. 2b and 3Sb).

**Fig. 4:**
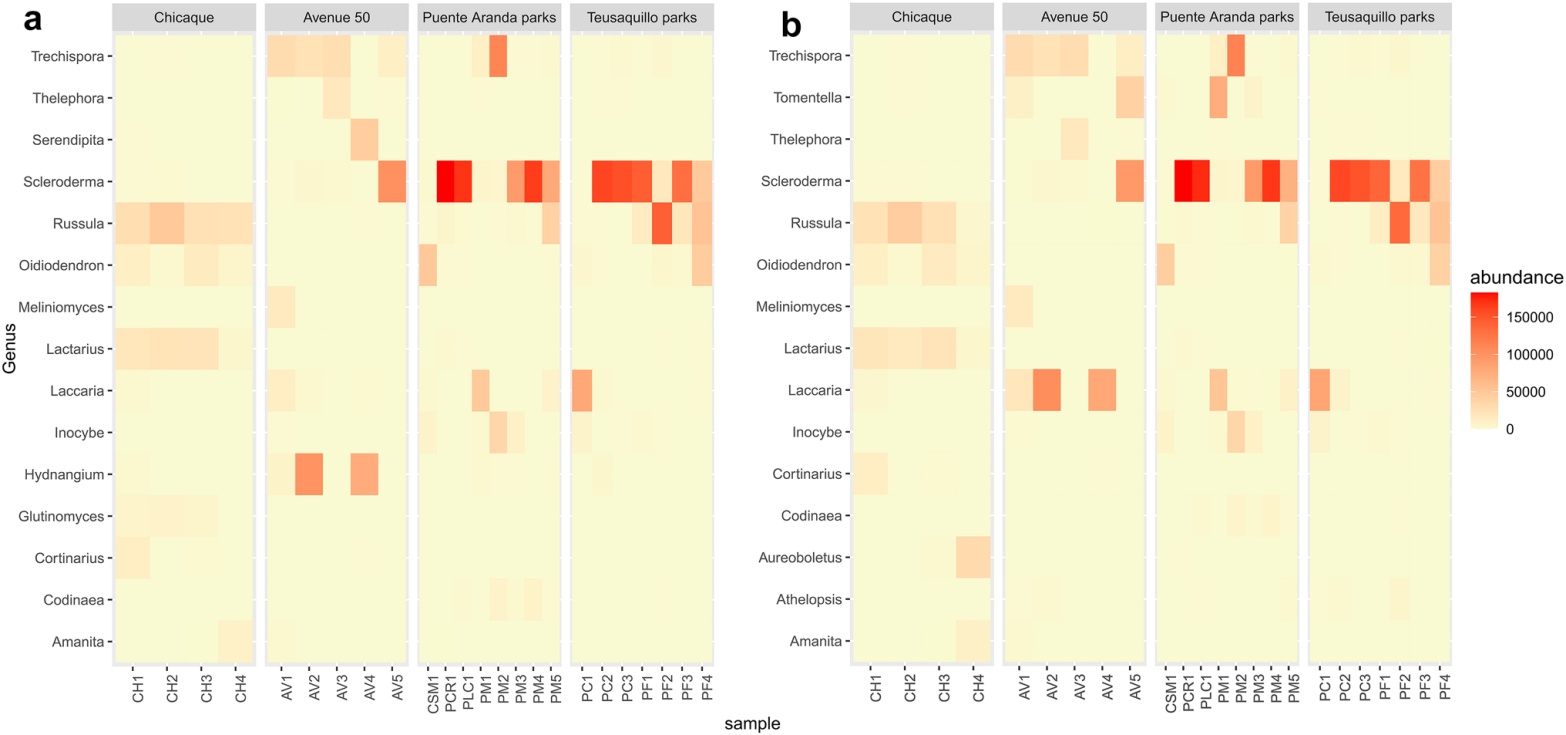
Heatmap based on genera abundance classified by **a** OTUs and **b** ASVs

An analysis of OTUs from urban samples using FungalTraits (Põlme et al. 2020) to determine their primary lifestyle shows that most reads belong to ECM fungi (66% of the total number of reads) followed by saprotrophs (12%) and root endophytes (2%). Also, 19% of the total number of reads belonged to OTUs that have not been classified within any functional group and were labeled as “unknown” (Fig. 3a). Similarly, rural samples show that ECM was the primary lifestyle with the higher percentage of reads (54%) followed by saprotroph (12%) and root endophytes (5%). A 29% of the total number of reads were also classified as “unknown” (Fig. 3b). A similar pattern was found for ASV data (Fig. 4S). For urban samples, most reads were classified as ECM fungi (70%) followed by saprotrophs (12%), root endophytes (1%), and 15% of “unknown” (Fig. 4Sa). Meanwhile, in rural samples, 60% were ECM fungi followed by saprotroph (13%) and 26% of “unknown” (Fig. 4Sb). The root endophyte percentage for rural samples was slightly lower for ASVs (< 1%) compared to the discovered using OTUs.

**Fig. 5:**
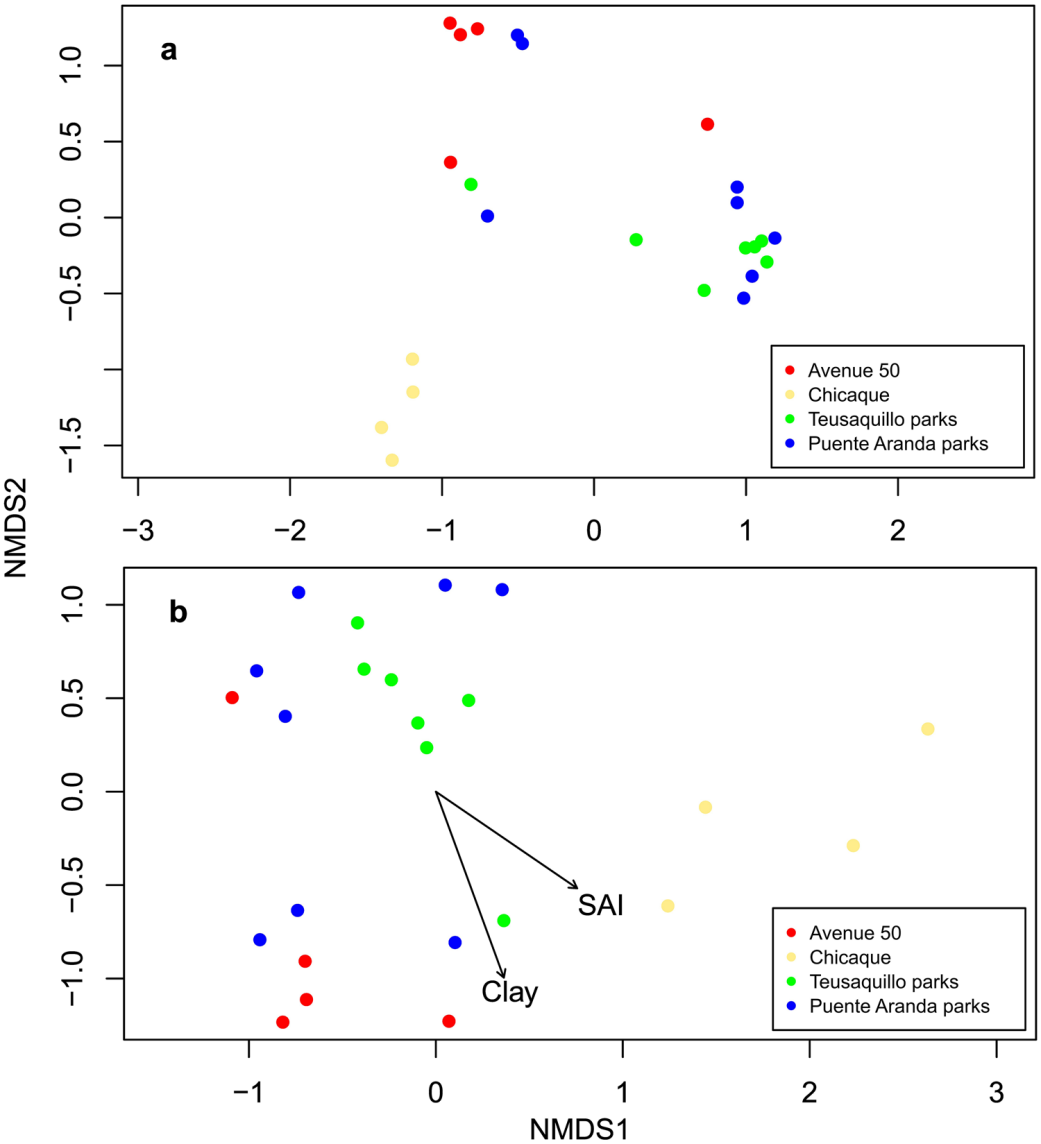
Nonmetric multidimensional scaling (NMDS) of fungal communities using **a** OTUs data and **b** ASVs data with significant soil variables correlated with the ordination. Avenue 50 (*n* = 5), Puente Aranda parks (*n* = 8), Teusaquillo parks (*n* = 7), Chicaque (*n* = 4)

The most abundant genera for the urban sites, based on both OTUs and ASVs, were *Scleroderma* for Puente Aranda and Teusaquillo parks and *Hydnangium* and *Trechispora* for Avenue 50, while the most abundant genera for the rural site in Chicaque were *Russula* and *Lactarius*. Relative abundances for the 15 most abundant genera for all sites based on the OTUs and ASV classification are found in Fig. 4. Also, bar plots for genera and families are shown in the Supplementary Material (Figs. 5S and 6S) which also show evidence of the great differences in composition between rural and urban sites, particularly the high abundance of *Scleroderma* in Puente Aranda and Teusaquillo parks.

The NMDS and ADONIS analysis, using again both OTUs and ASVs, showed a clear separation of the samples from the rural site in Chicaque from all the urban sites (Table 2; Fig. 5). Urban samples from the Avenue 50 site were also significantly different in species composition from the urban parks in Teusaquillo and Puente Aranda (Table 2; Fig. 5).

**Table 2:** ADONIS analysis using OTUs and ASVs data

	**Chicaque**		**Avenue 50**		**Puente Aranda parks**	
	OTUs	ASVs	OTUs	ASVs	OTUs	ASVs
**Avenue 50**	0.032*	0.028*	-	-	-	-
**Puente Aranda parks**	0.018*	0.02*	0.058^a^	0.042*	-	-
**Teusaquillo parks**	0.018*	0.028*	0.032*	0.006*	0.587	0.083

Sites showing significant differences (*p* < 0.05) are marked with "*". *P*-values between 0.05 and 0.07 were considered marginally significant and are marked with "a". Adjustment method for multiple comparisons: holm

Even though there were significant differences in soil variables among sites and species composition independently, ordination based on OTUs was not significantly correlated with any of the soil variables measured in this study (Fig. 5a). However, we observed that the percentage of clay (*p* = 0.046) and percent of exchangeable acidity saturation (SAI, *p* = 0.079) were found to be significantly and marginally significantly correlated with the NMDS ordination based on ASV data (Fig. 5b).

## Discussion

This study explored the differences in diversity and species composition of root-associated fungal communities of the tree *Q. humboldtii* when growing in urban ecosystems of Bogotá and nearby rural ecosystem of the Chicaque natural park. This comparison allows the identification of biotic and abiotic variables that may be important in structuring ECM fungal communities in tropical urban systems.

### Comparison between OTUs and ASVs community patterns

In general, the results between OTUs and ASVs were very similar, and no substantial differences were observed between the community patterns of root-associated fungi obtained with both bioinformatics pipelines. Alpha diversity patterns were the most affected by the bioinformatic pipeline used. When using OTUs, the sampling effort was sufficient to reach a plateau in the species accumulation curves, whereas with ASVs, the sampling effort was not enough to reach a plateau. This difference is due to intrinsic differences in the methods; OTUs are based on clusters of similar sequences within a representative group, while ASVs are based on unique sequences. However, results of the diversity indices, NMDS, and classification of functional groups did not differ much between OTUs and ASVs. Depending on the method used, some differences were found in the top 15 most abundant families and genera. For example, *Tomentella* was excluded from the top 15 when using OTUs, but was included when using ASVs. However, both methods showed the same community patterns and coincided in most of the abundant families and genera for all sampling sites.

### Differences in root-associated fungal communities between rural and urban sites

The number of samples taken in each sample site was relatively similar, allowing adequate comparisons to be made between sites. However, the uneven number of samples between rural and the whole set of urban sample sites could lead to lower representativeness of rural fungal communities that must be taken into account when comparing with the urban sample set communities. We found significant differences in the species richness between rural and urban samples when using the Shannon index but not by the Alpha Fisher index that showed a high degree of variability (Fig. 2). The species accumulation curves per site (Fig. 7S) did not show clear differences between individual sites probably due to low replication, coupled to a high variability in the ECM communities. The non-homogeneity between sampled sites could have led to a biased representation of the rural communities when compared to the whole set of urban samples. Previous studies from temperate ecosystems had found significant differences in species richness between rural and urban communities. For example, Baxter et al. (1999) studied forest stands dominated by *Quercus rubra* and *Q. velutina* in New York State (USA) and found a higher average richness of ECM fungi in rural samples compared to urban samples. Also, Ochimaru and Fukuda (2007) found that the diversity of ECM fungi in forests dominated by *Q. myrsinaefolia* was significantly higher in rural samples compared with urban and suburban samples in the Kanto District (Japan).

NMDS analysis showed that community composition of samples from the rural site in Chicaque was significantly different from all the urban sites. These differences were corroborated with the ADONIS results that showed Chicaque as a significantly different community (Table 2). In addition, clay % and SAI were correlated only with the ASV community composition, whereas the OTU ordination was not correlated with any of the environmental variables (Fig. 5). Studies from temperate sites have also found differences in the composition of the ECM fungal communities between urban and natural sites with pH, P, and ammonia being the soil variables with higher explanatory power for variation in species composition (Martinová et al. 2016; Scholier et al. 2023). In our case, soil texture (clay %) has been reported as an important determinant of certain soil parameters like SOM storage and stability that finally influence soil communities. For example, clay or clay-silt soil size particles intervene in SOM storage by modifying C fluxes and their rate of turnover and being the particles that domain the interaction of minerals with metals, organic compounds, water, or other minerals in soils (Rasmussen et al. 2018; Sposito et al. 1999; Wieder et al. 2015). In addition, some studies like Sessitsch et al. (2001) have reported that clay soil particles have a positive relation with the diversity and biomass of soil bacterial communities, which opens up the possibility that it may also be applicable to fungal communities. However, those hypotheses are far from being clear and applicable in all ecosystems or soil microorganisms like fungi, where more research is needed. It is important to highlight that soil variables are highly correlated with each other, making it difficult to separate the independent effect of each variable on the fungal community.

The analysis of soil variables from our study shows that Chicaque has a more acidic soil, lower C:N, higher SAI, and higher CEC compared with the urban sites (Table 1, Fig. 2Sd). Soil clay percentage and SAI are strongly related to soil pH and can affect the availability of certain soil nutrients. The exchangeable acidity saturation percent (SAI) refers to the sum of basic cations available for exchange in soil and in relation to the cation exchange capacity, which affects the hydrogen ions available in soil. Meanwhile, clay soils have a smaller particle size and a higher cation exchange capacity. Soil pH has been shown to influence fungal communities acting directly on fungi or indirectly by affecting plant diversity and composition (Tedersoo et al. 2020). Therefore, we believe soil variables related to soil pH are clearly differentiating urban fungal communities from rural ones and also potentially between the two urban clusters in our ASVs ordination.

The lack of correlation between the soil variables measured and the NMDS based on OTUs data could be associated with the low replication at this site and also by the fact that the composition of ECM fungal communities has been shown to be affected by multiple environmental factors that were not measured in this study such as water availability, temperature, and anthropogenic factors such as deposition of N or heavy metals and other pollutants (like CO, O_3_, SO_2_, NO_2_), urbanization, heat islands, and edge effects (Baruch et al. 2020; Jumpponen et al. 2010; Karpati et al. 2011; Martinová et al. 2016). In addition, historical land use of the sites and habitat fragmentation induced by urbanization can result in different fungal communities by limiting spore dispersal or by introductions by humans through cultivation/agricultural practices (Baruch et al. 2020; Karpati et al. 2011). According to the silvicultural management guide for Bogotá, urban trees are fertilized with high concentrations of P and K in their first year of transplantation and later with fertilizers rich in N to favor the development of roots and foliage (Mahecha et al. 2010). In addition to fertilization, disturbances such as cultivation, gardening, and watering that are often applied to urban trees in Bogotá can impact mycelial networks and species interactions (Baruch et al. 2020; Köhl et al. 2014).

Biotic disturbances could also affect ECM communities in urban systems. *Quercus humboldtii* is a tropical monodominant species that usually grows in high densities hosting high density populations of its associated ECM fungi. However, in urban sites, trees are usually scattered probably affecting the fungal population dynamics and fungal interactions with biotic and abiotic factors (Baruch et al. 2020). In addition, the introduction of exotic plants such as *Pinus* spp., *Eucalyptus* spp., and *Acacia* spp. with their associated fungi can disrupt fungal communities of native species particularly if growing in close proximity as is often the case in Bogotá. For this reason, it is important to continue to investigate the biotic or abiotic factors structuring communities of ECM fungi in urban ecosystems.

The high abundance of *Scleroderma* found in urban sites is consistent with the results of previous studies. Karpati et al. (2011) worked in a metropolitan area of New Jersey (USA) comparing undisturbed forests and disturbed urban sites. These authors found that *Scleroderma* was present in all urban sites, suggesting that this genus was typically associated with disturbed ecosystems. Also, consistent with our results, *Russula* and *Lactarius* have been found to be good indicators of natural ecosystems illustrated by their high abundance in undisturbed sites (Karpati et al. 2011; Martinová et al. 2016). A high number of ECM species of the Russulaceae family have been found in several rural sampling sites in Colombia based on fruiting body surveys (Vargas and Restrepo 2020).

Fruiting bodies collected near *Q. humboldtii* trees sampled in our urban sites were identified based on morphology and the ITS region as *Scleroderma bovista* (GenBank accession number: SUB14173651). Many species of the genus *Scleroderma* are used as inoculants for commercial plantations of various tree species because of their ability to form ectomycorrhizas with many plants and to protect their host plants against pathogens (Pinzón-Osorio and Pinzón-Osorio 2018). *Scleroderma bovista* has a wide distribution, with several reports in Europe and the Americas, this species being reported in urban and rural areas of Colombia as an ectomycorrhizal species (Pinzón-Osorio and Pinzón-Osorio 2018). We speculate that the high abundance of *Scleroderma* on urban trees could be caused by inoculation of urban trees with exotic species used as inoculant. This hypothesis will need to be tested in future studies. The genus *Laccaria* is a typical ECM taxon reported in plant communities on almost all continents (Kropp and Mueller 1999). Species in the genus *Laccaria* have been reported as pioneer species, frequently found in disturbed sites (Kropp and Mueller 1999), and have also been reported as nitrophilic (associated to high nitrogen conditions) in temperate and tropical forests (Corrales and Ovrebo 2020; Lilleskov et al. 2011). Given these previously reported functional characteristics, it is not surprising to find it at high abundances in our urban sampling sites (Fig. 4).

## Conclusions

In Colombia, the species composition and richness patterns of ECM and other root-associated fungi are poorly studied, and so far, very little research has focused on fungi associated with urban environments. We found that the fungal community composition is significantly different between urban and rural samples and that soil variables that influence soil pH (like cation exchange capacity, soil texture, and exchangeable acidity saturation percent) could be important drivers of that trend. However, other factors including tree age or management practices could also influence fungal community composition. Finally, the use of OTUs or ASVs did not result in different outcomes when studying communities of root-associated fungi particularly if used for community composition analysis. ASVs cannot be recommended for estimation of species richness due to potential overestimation.

### Supplementary Information

Below is the link to the electronic supplementary material.

Supplementary file1 (DOCX 990 kb)

## Data Availability

All HTS data associated with this work is available through the NCBI SRA PRJNA1063376.
