# Supplementary material for: Ectomycorrhizal fungal communities in natural and urban ecosystems: Quercus humboldtii as a study case in the tropical Andes
Source: Mycorrhiza. 2024 Mar 14;34(1-2):45–55. doi: 10.1007/s00572-024-01140-0 (PMC10998789; doi:10.1007/s00572-024-01140-0)
Supplement: Supplementary file 1 — Supplementary file1 (DOCX 990 kb) [file 572_2024_1140_MOESM1_ESM.docx]

**Supplementary material**

Journal: Mycorrhiza

**Variation in ectomycorrhizal fungal communities between natural and urban ecosystems: *Quercus humboldtii* as a study case in the tropical Andes**

Juan David Sanchez Tello^1^ and Adriana Corrales^1,2^

^1^ Center for Research in Microbiology and Biotechnology-UR (CIMBIUR). Faculty of Natural Sciences. Universidad del Rosario. Bogotá. Colombia

^2^ Society for the Protection of Underground Networks, SPUN, 3500 South DuPont Highway, Dover, DE 19901, USA

Address for manuscript correspondence:

juandavi.sanchez@urosario.edu.co

juandasanchez061@gmail.com

ORCID Juan David Sanchez Tello: 0009-0006-7040-300X

ORCID Adriana Corrales: 0000-0001-9885-4634


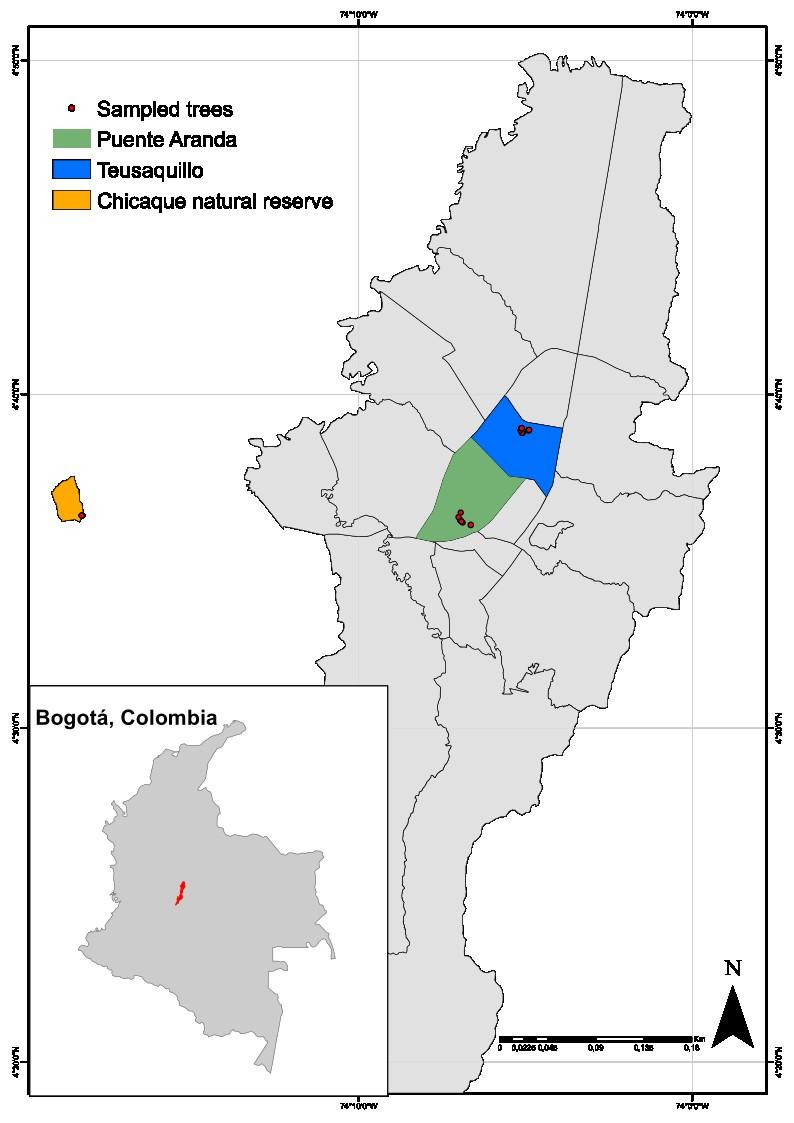


**Fig. 1S** Location of sampling sites. Rural sites were located in Chicaque Natural Reserve, and urban sites were in the districts of Puente Aranda and Teusaquillo (which contains the Avenue 50 and Teusaquillo parks samples) (Bogotá. Colombia)


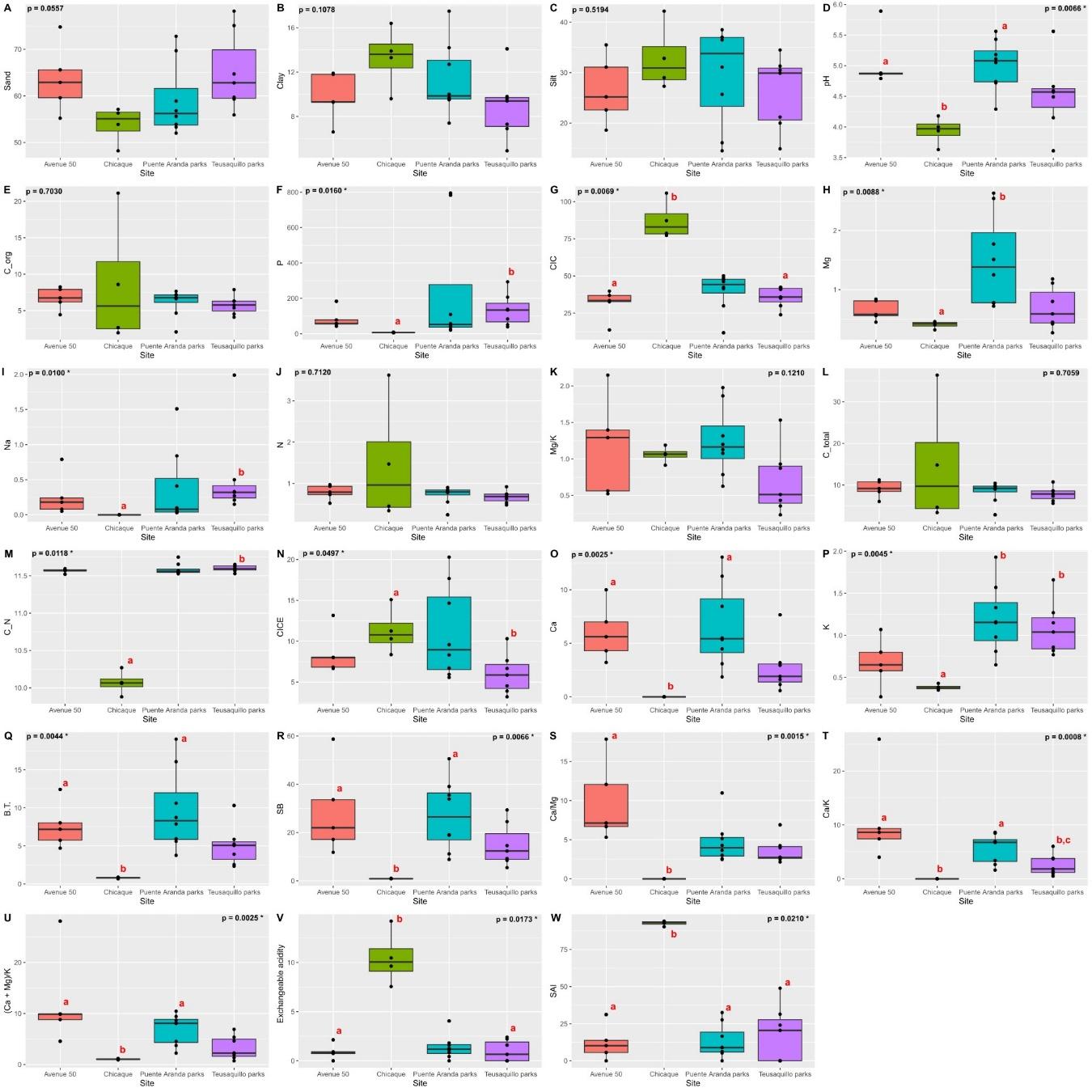


**Fig. 2S** Boxplots of analyzed soil variables per site. Significant differences are represented by a * (p <0.05) and red letters. Avenue 50 (n=5), Puente Aranda parks (n=8), Teusaquillo parks (n=7), Chicaque (n=4)


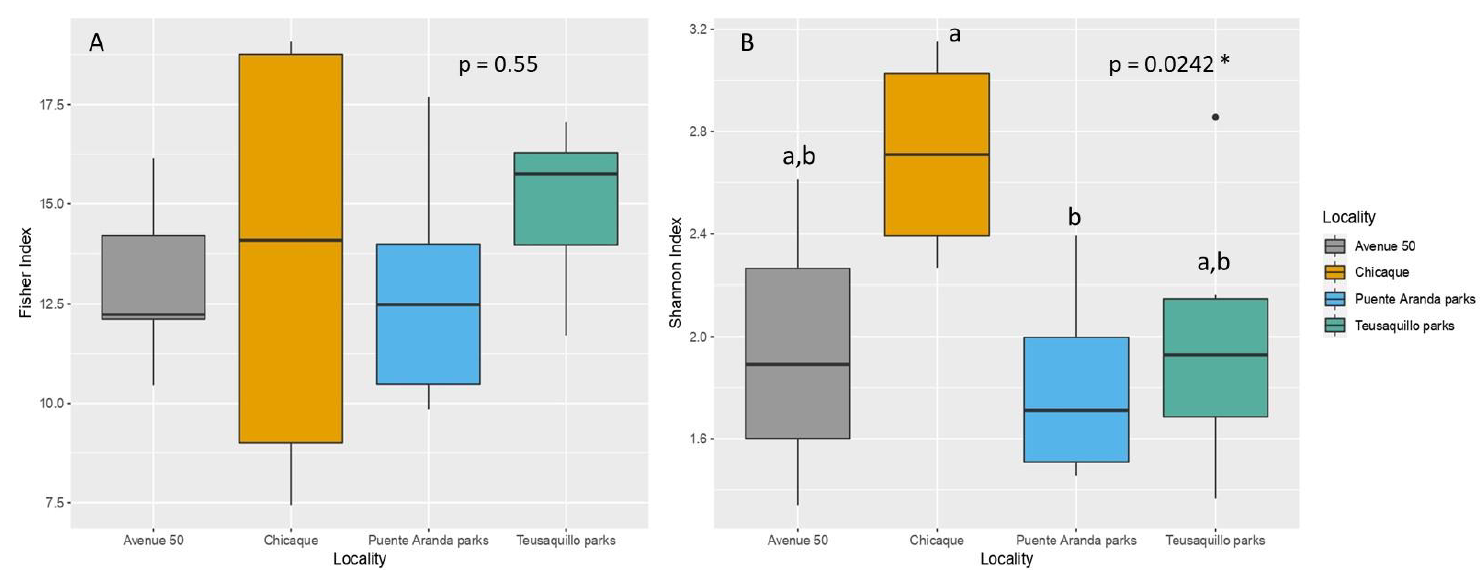


**Fig. 3S** Alpha diversity indices boxplots for ASVs. A) The Alpha Fisher diversity index does not show significant differences, and B) the Shannon index shows significant differences between Chicaque and Puente Aranda Parks. Significant differences are represented by a * (p <0.05). Avenue 50 (n=5), Puente Aranda parks (n=8), Teusaquillo parks (n=7), Chicaque (n=4)

**
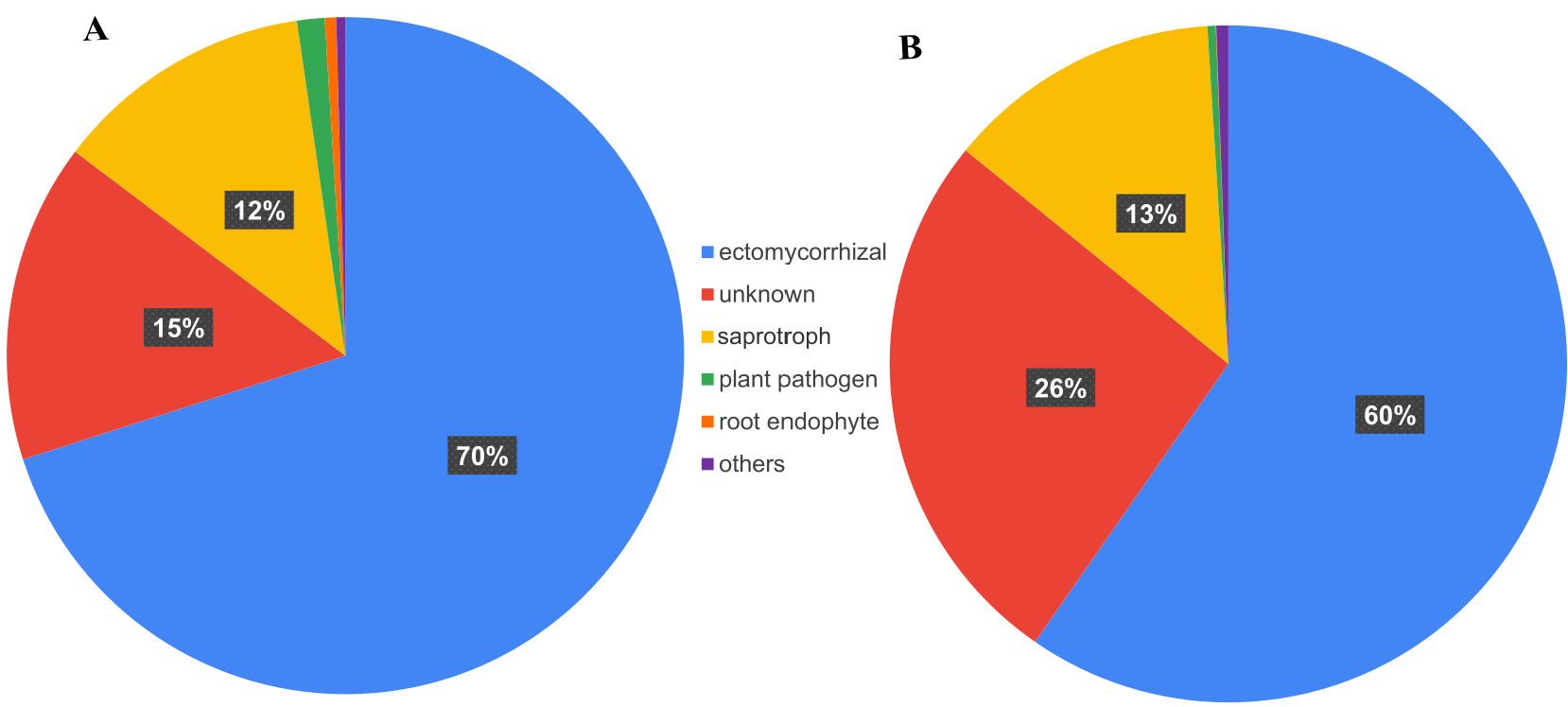
Fig. 4S** Classification of ASVs primary lifestyle classification based on the FungalTraits database for A) urban samples, and B) rural samples

**
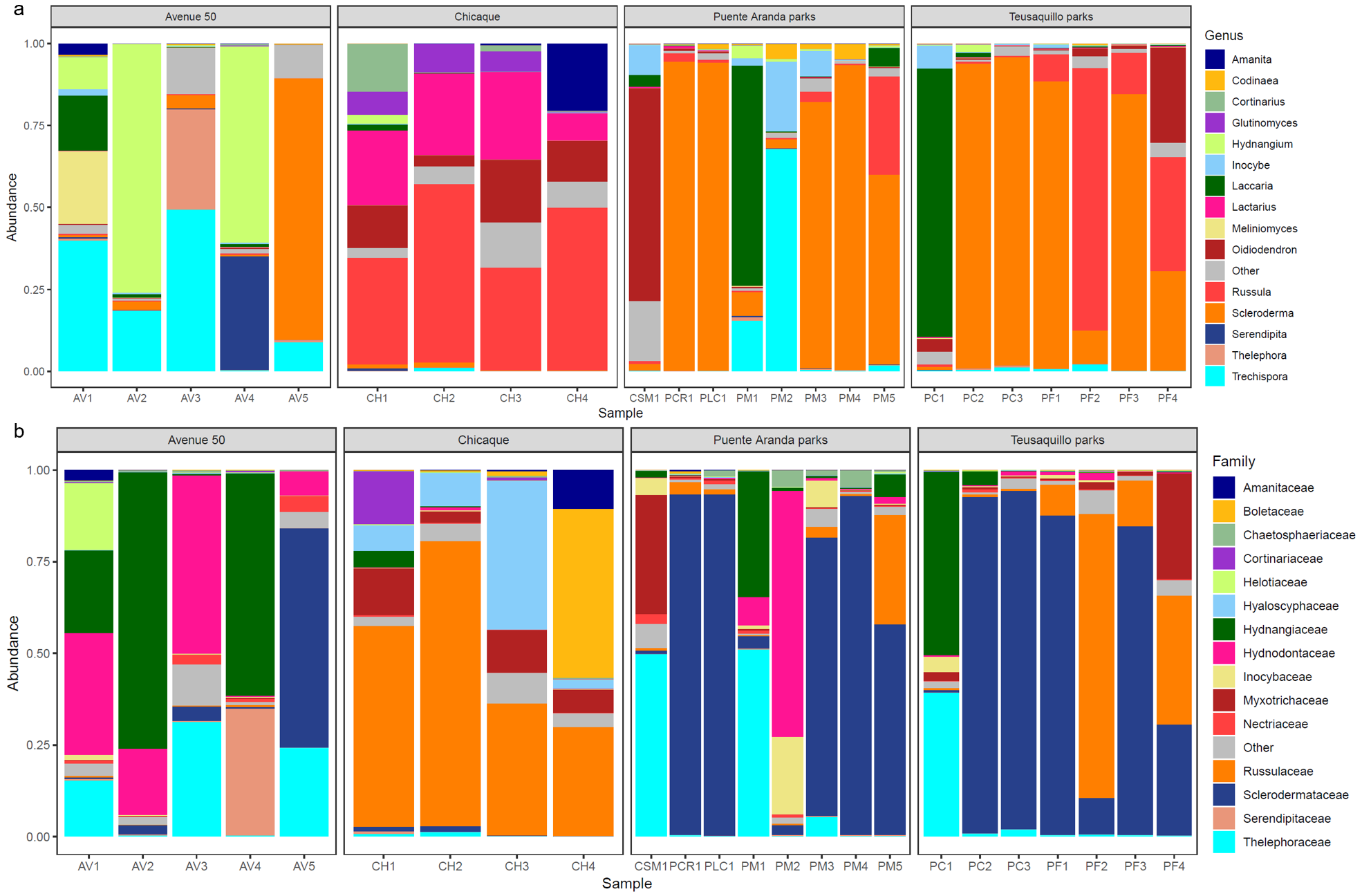
**

**Fig. 5S** Genus and family abundance bars classified by OTUs. a) Top 15 most abundant genera. b) Top 15 most abundant families

**Fig. 6S** Abundance bars of the top 15 most abundant genera and families classified by ASVs
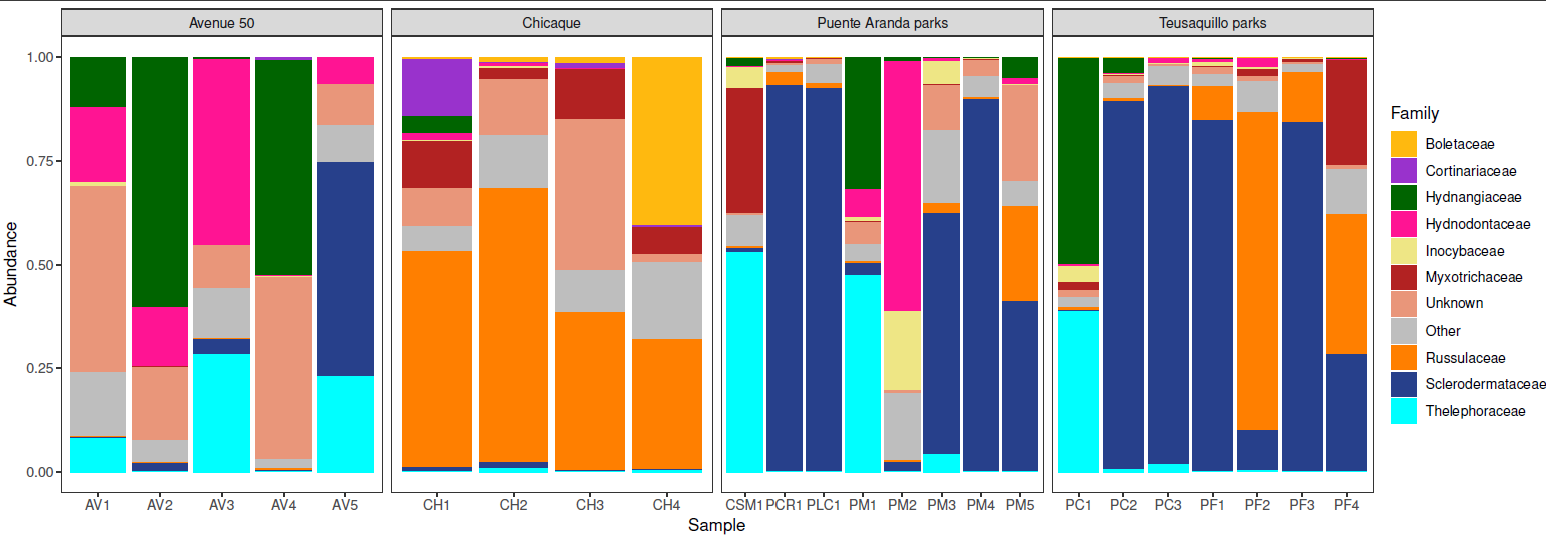

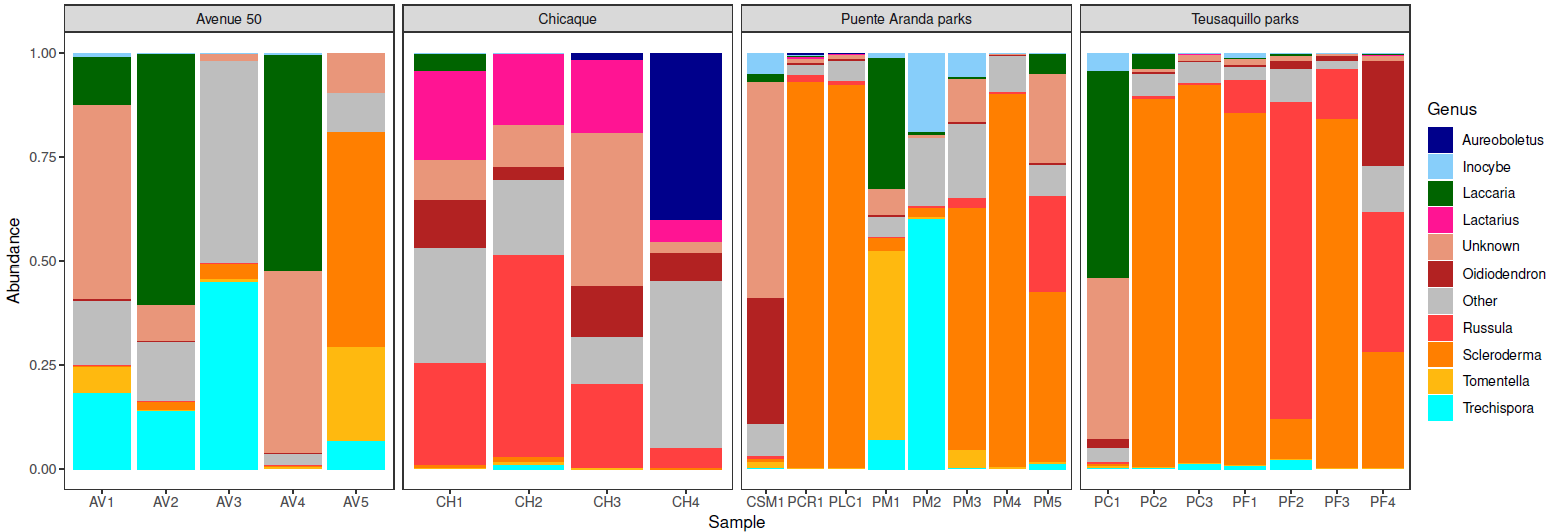


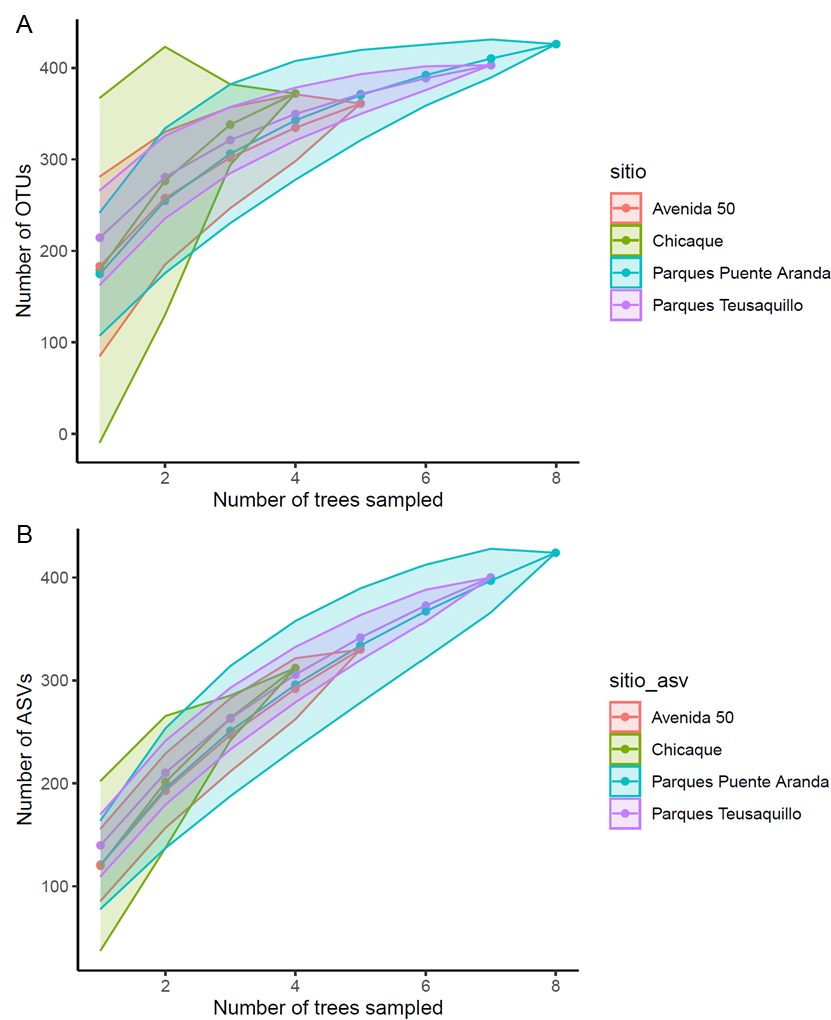


**Fig. 7S** Accumulation curves per site for A) OTUs data and B) ASV data


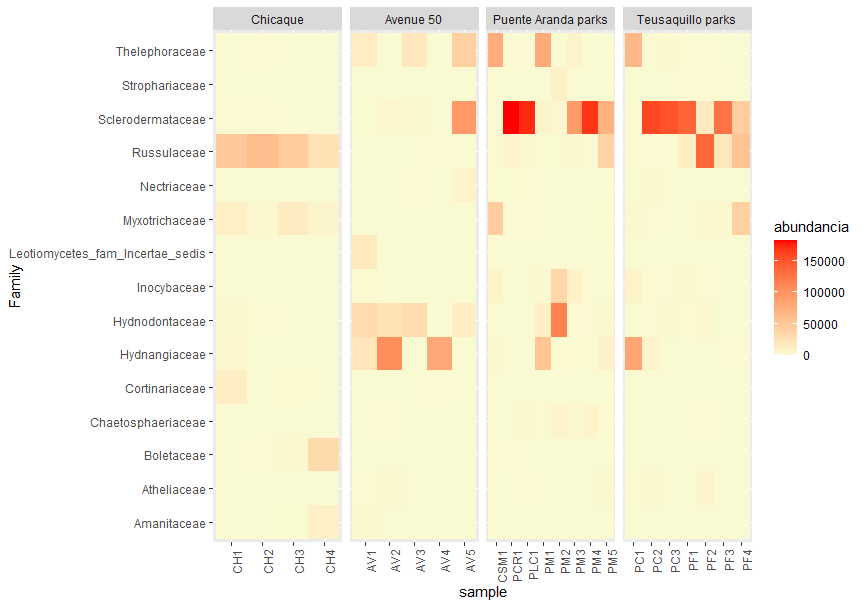


**Fig. 8S** Heatmap of family abundance classified by ASVs


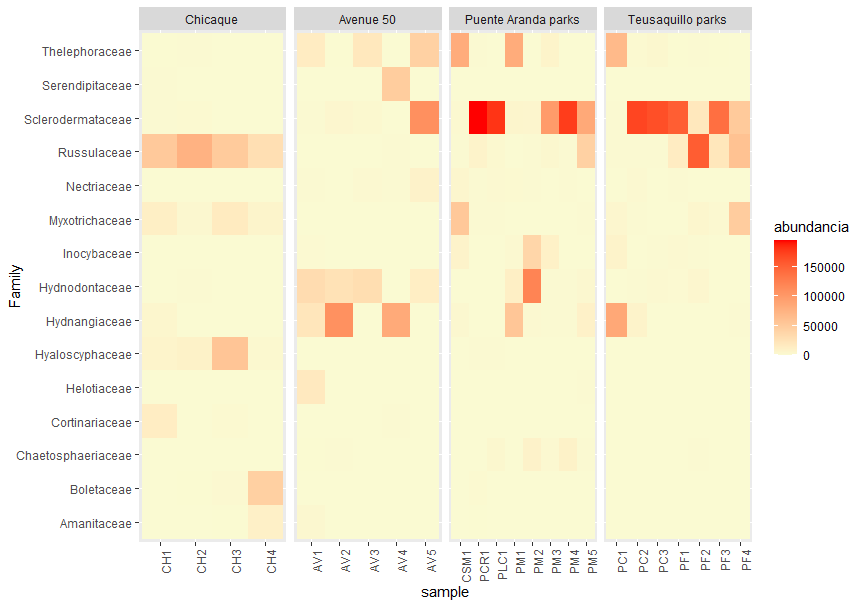


**Fig. 9S** Heatmap of family abundance classified by OTUs
